# Extracellular matrix-derived and low-cost proteins to improve polyurethane-based scaffolds for vascular grafts

**DOI:** 10.1038/s41598-022-09040-z

**Published:** 2022-03-28

**Authors:** Isabella C. P. Rodrigues, Éder S. N. Lopes, Karina D. Pereira, Stephany C. Huber, André Luiz Jardini, Joyce M. Annichino-Bizzacchi, Augusto D. Luchessi, Laís P. Gabriel

**Affiliations:** 1 School of Applied Sciences, University of Campinas, Rua Pedro Zaccaria, 1300, Limeira, SP 13484-350 Brazil; 2 School of Mechanical Engineering, University of Campinas, Rua Mendeley, 200, Campinas, SP 13083-860 Brazil; 3 Institute of Biosciences, São Paulo State University, Rio Claro, SP Brazil; 4 Hematology and Hemotherapy Center, University of Campinas, Campinas, SP Brazil; 5 School of Chemical Engineering, University of Campinas, Campinas, SP Brazil

**Keywords:** Cardiology, Materials science

## Abstract

Vascular graft surgeries are often conducted in trauma cases, which has increased the demand for scaffolds with good biocompatibility profiles. Biodegradable scaffolds resembling the extracellular matrix (ECM) of blood vessels are promising vascular graft materials. In the present study, polyurethane (PU) was blended with ECM proteins collagen and elastin (Col-El) and gelatin (Gel) to produce fibrous scaffolds by using the rotary jet spinning (RJS) technique, and their effects on in vitro properties were evaluated. Morphological and structural characterization of the scaffolds was performed using scanning electron microscopy (SEM) and atomic force microscopy (AFM). Micrometric fibers with nanometric rugosity were obtained. Col-El and Gel reduced the mechanical strength and increased the hydrophilicity and degradation rates of PU. No platelet adhesion or activation was observed. The addition of proteins to the PU blend increased the viability, adhesion, and proliferation of human umbilical vein endothelial cells (HUVECs). Therefore, PU-Col-El and PU-Gel scaffolds are promising biomaterials for vascular graft applications.

## Introduction

Cardiovascular diseases are the leading cause of death worldwide, with more than 18.5 million deaths registered in 2019^1^. Vascular graft surgery is often required to repair blood vessel obstruction or damage in patients with cardiovascular disease. Limitations of autografts and allografts, such as hyperplasia and inflammation^2^, have encouraged the use of synthetic grafts as scaffolds for vascular replacement or repair using tissue engineering techniques. Ideal vascular grafts should have favorable biodegradation properties to avoid long-term complications. Biodegradable synthetic polymers, including polylactic acids, polyglycolic acids, polycaprolactones, and polyurethanes, are used as vascular grafts in practice^3^. Among these, polyurethanes (PUs) are particularly flexible, hemocompatible, and mechanically stable polymers that are widely used in vascular applications^4^.

Scaffolds resembling the extracellular matrix (ECM) of blood vessels are promising alternatives to achieve favorable biocompatibility profiles. ECM proteins, including different types of collagens, elastin, gelatin, and other glycoproteins, have been successfully used to obtain such scaffolds. Collagen (Col) and elastin (El) are the main proteins in the ECM of blood vessels. Type I collagen is a fibrous protein that is responsible for strength and structural support, whereas elastin provides elasticity and resilience to the ECM^5^. However, expensive extraction procedures are typically required to obtain these proteins^6^. To this end, gelatin (Gel) may be used as an alternative highly biocompatible protein that can be obtained from denatured collagen at a lower cost^7,8^. These features make Gel an outstanding alternative for the development of high-performance vascular grafts.

While using proteins in vascular grafts increases the biocompatibility of the scaffold, studies have also demonstrated relatively high degradation rates and low mechanical integrity of these materials^9^. A possible strategy to improve this is blending proteins with synthetic polymers, such as PUs. The compatibility with endothelial cells (ECs) and the anti-thrombogenic properties of such scaffolds must also be evaluated. ECs play an essential role in blood vessel physiology by preventing thrombosis and hyperplasia, and promoting hemostasis and nutrient exchange^10^. Acellular or cell-cultured scaffolds should therefore promote adhesion and proliferation of ECs. Many factors can affect the interactions between scaffolds and ECs, such as the type of materials, cell morphology, hydrophilicity, and other nanometric features^10,11^. Moreover, the scaffold should not cause thrombosis; therefore, assessing the association between scaffolds and blood components, such as platelets, is of utmost importance before conducting in vivo assessments^12^.

Various methods have been explored for the production of vascular and other soft tissue-engineered scaffolds. Fiber production methods are attractive because of the ability of fibrous scaffolds to mimic the ECM structure^13^. Rotary jet spinning (RJS) is a novel fiber production method that allows high production rates at low costs^14^. Unlike the well-known electrospinning method, RJS does not require high voltage, and protein denaturation is thus avoided^15,16^. We also previously showed that RJS can be used to produce tubular scaffolds with a diameter of less than 6 mm, which is advantageous for coronary applications^17^.

In our previous studies, we investigated the optimum parameters and intrinsic properties of PU-based scaffold production using RJS^17–19^. In the present study, we hypothesized that the addition of ECM-derived proteins to PU would improve the properties of RJS vascular graft scaffolds. We fabricated RJS PU, PU-Col-El, and PU-Gel and characterized the in vitro effects of these scaffolds. The effect of blending PU with different proteins was evaluated by determining the scaffold resistance, degradation, hydrophilicity, and the ability to interact with ECs and platelets under physiological conditions. The RJS scaffolds proposed here may serve as innovative vascular graft alternatives.

## Results

### Fabrication and morphological characteristics of scaffolds

**Figure 1:**
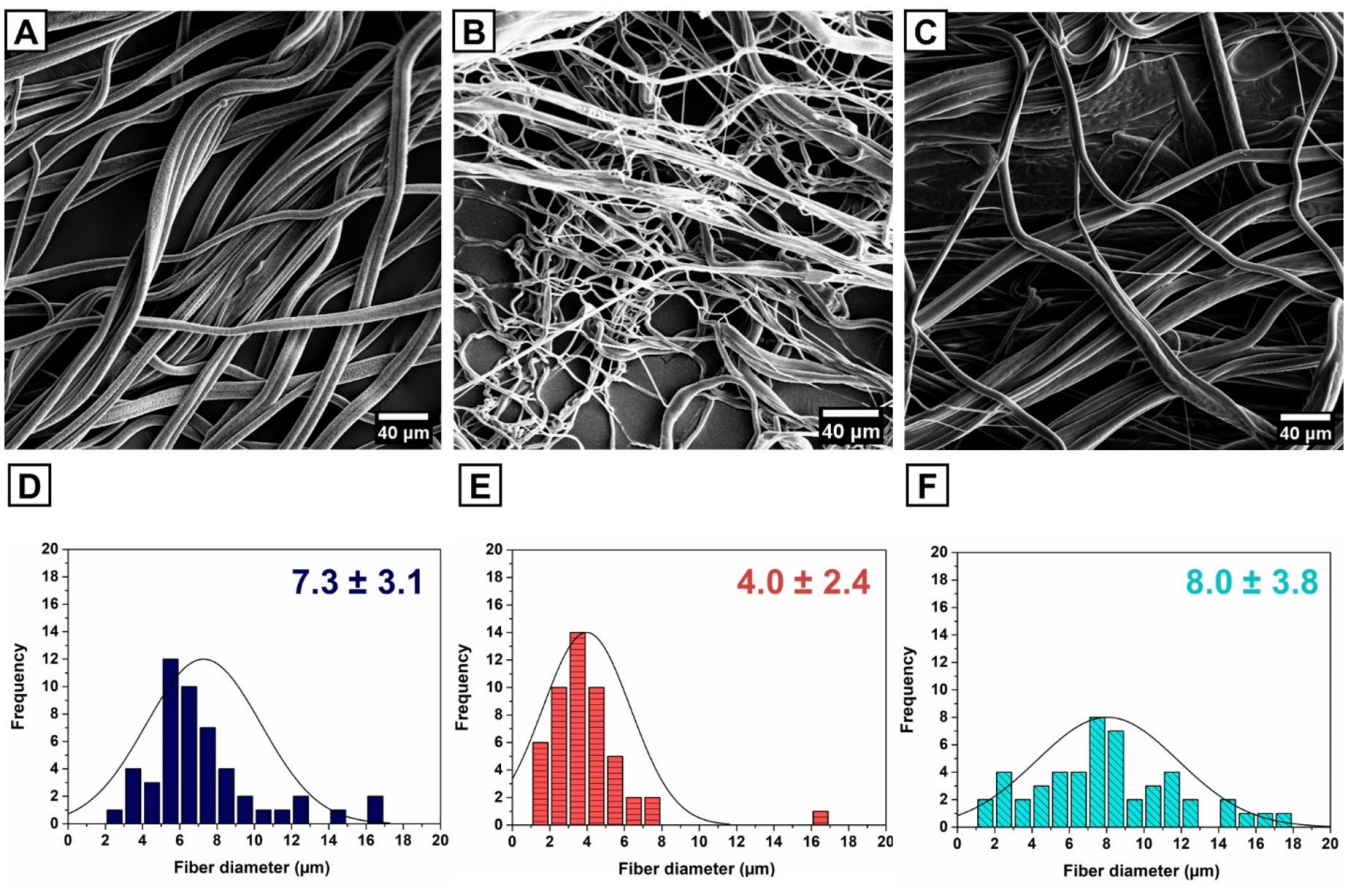
SEM images and fiber diameter distributions of PU (**A**, **D**), PU-Col-El (**B**, **E**), and PU-Gel (**C**, **F**) scaffolds.

PU, PU-Col-El, and PU-Gel scaffolds were fabricated using the RJS method, and their morphologies were visualized using scanning electron microscopy (SEM) (Fig. 1A–C). All scaffolds presented homogenous and bead-free fibers with diameters of up to 18 µm. The PU-Col-El scaffolds included fibers with smaller diameters, and a narrower range than the PU and PU-Gel scaffolds (Fig. 1D–F). The smaller average diameter was due to the high rotational speed needed to produce homogenous fibers for PU-Col-El scaffolds, which has been previously reported^17^.

**Figure 2:**
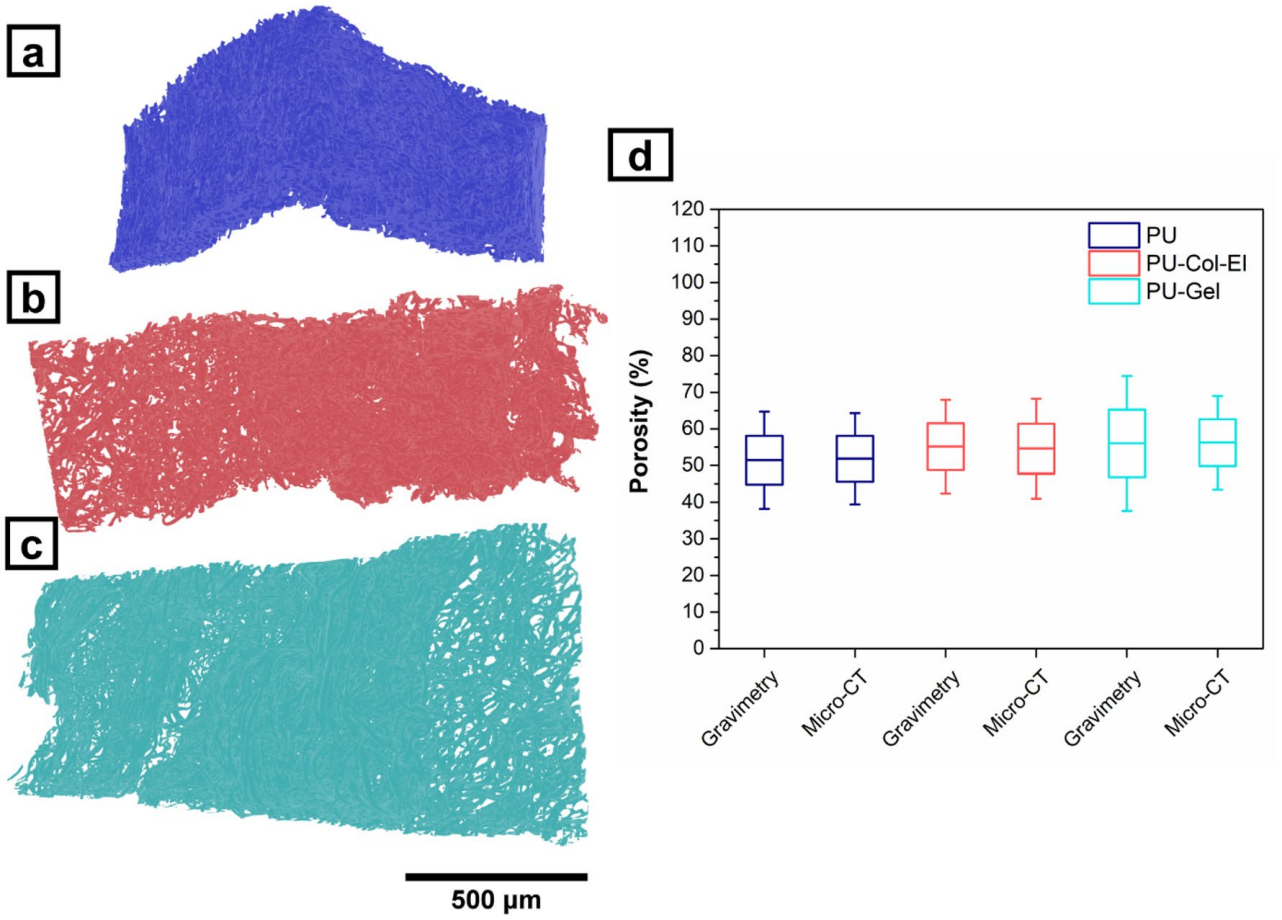
Micro-CT images of PU (**a**), PU-Col-El (**b**), and PU-Gel (**c**) scaffolds and porosity results obtained by micro-CT and gravimetry (**d**).

Microcomputed tomography (Micro-CT) provided further insight about the scaffolds’ microstructure and porosity (see Supplementary Video 1–3 online). Figure 2a–c shows three-dimensional (3D) models of scaffolds created from 2D slice images. PU, PU-Col-El, and PU-Gel scaffolds presented porosity of 51.9% ± 6.2%, 54.6% ± 6.8%, and 56.2% ± 6.4%, respectively. Considering that micro-CT analysis also include closed pores^20^, gravimetry was conducted using a liquid intrusion method to confirm the porosity results and pore interconnectivity. Figure 2d presents a comparison of porosity results for all scaffolds using micro-CT and gravimetry. The percent porosity obtained through gravimetry for PU, PU-Col-El, and PU-Gel scaffolds was 51.4% ± 6.6%, 55.2% ± 6.4%, and 56.0% ± 9.2%, respectively. There was no significant difference (p > 0.05) in porosity between scaffolds and techniques. This confirms that porous scaffolds with interconnected pores were obtained.

**Figure 3:**
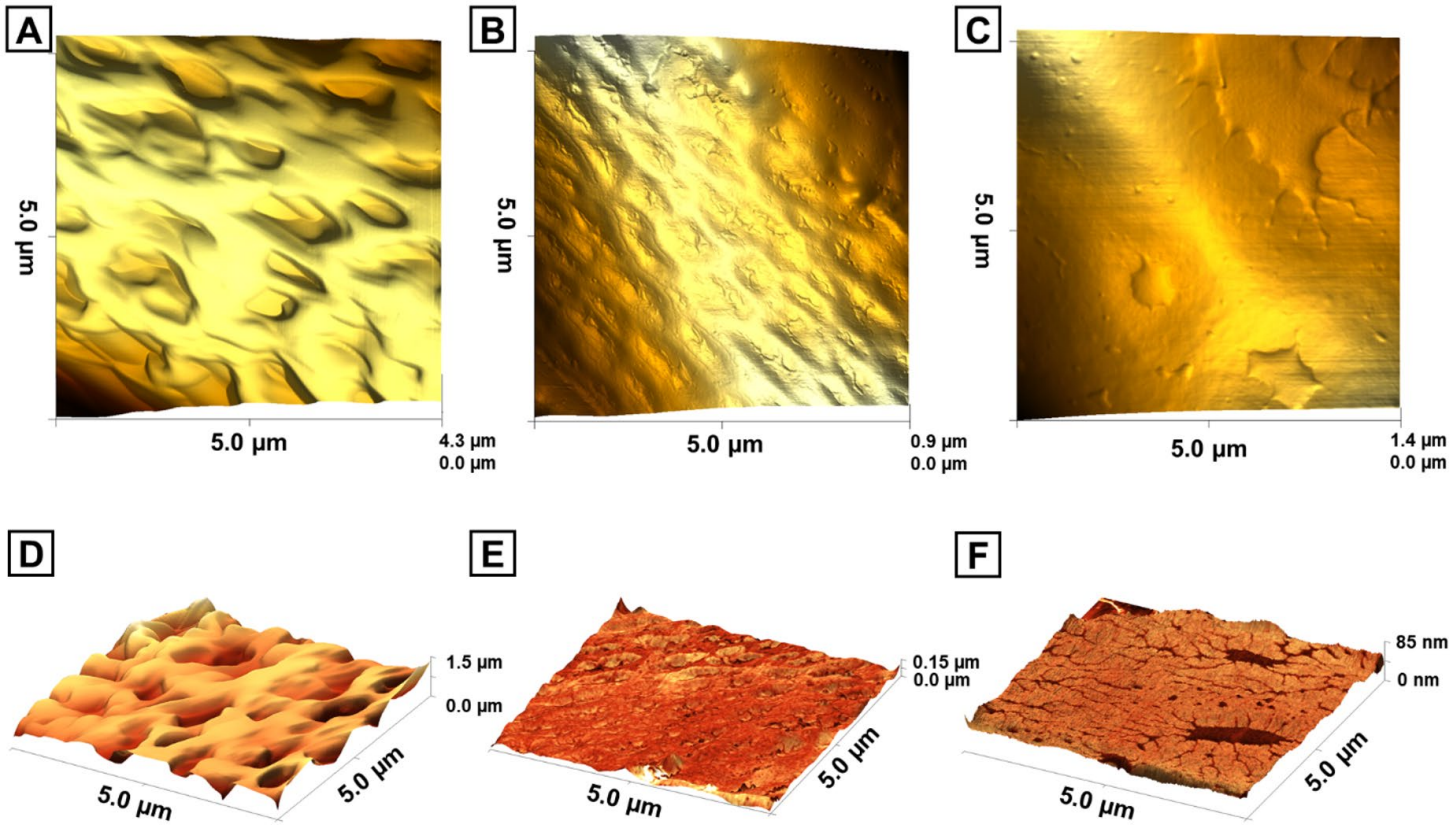
3D images obtained from AFM represented by a height model (top) and simultaneously acquired height and phase models (bottom) for PU (**A**, **D**), PU-Col-El (**B**, **E**), and PU-Gel (**C**, **F**) scaffolds.

Atomic force microscopy (AFM) was used to evaluate the surface morphologies of the produced fibers in intermittent contact mode. The 3D topographies of the fibers (Fig. 3A–C) revealed differences in the surface morphology of the scaffolds. Height models were combined with phase imaging to assess fiber roughness and identify regions with varying fiber properties (Fig. 3D–F). PU, PU-Col-El, and PU-Gel scaffolds had average roughness (Ra) levels of 162.9 ± 16.4 nm, 26.0 ± 8.0 nm, and 30.6 ± 11.3 nm, respectively. PU fibers yielded the highest level of roughness, whereas the addition of proteins resulted in smoother fiber surfaces in PU-Col-El and PU-Gel scaffolds. Some contrast areas from phase imaging can also be observed on the PU-Gel scaffold (Fig. 3F). These are likely gelatin deposits on the PU matrix surface.

### Wettability, degradation, and mechanical profiles of scaffolds

**Figure 4:**
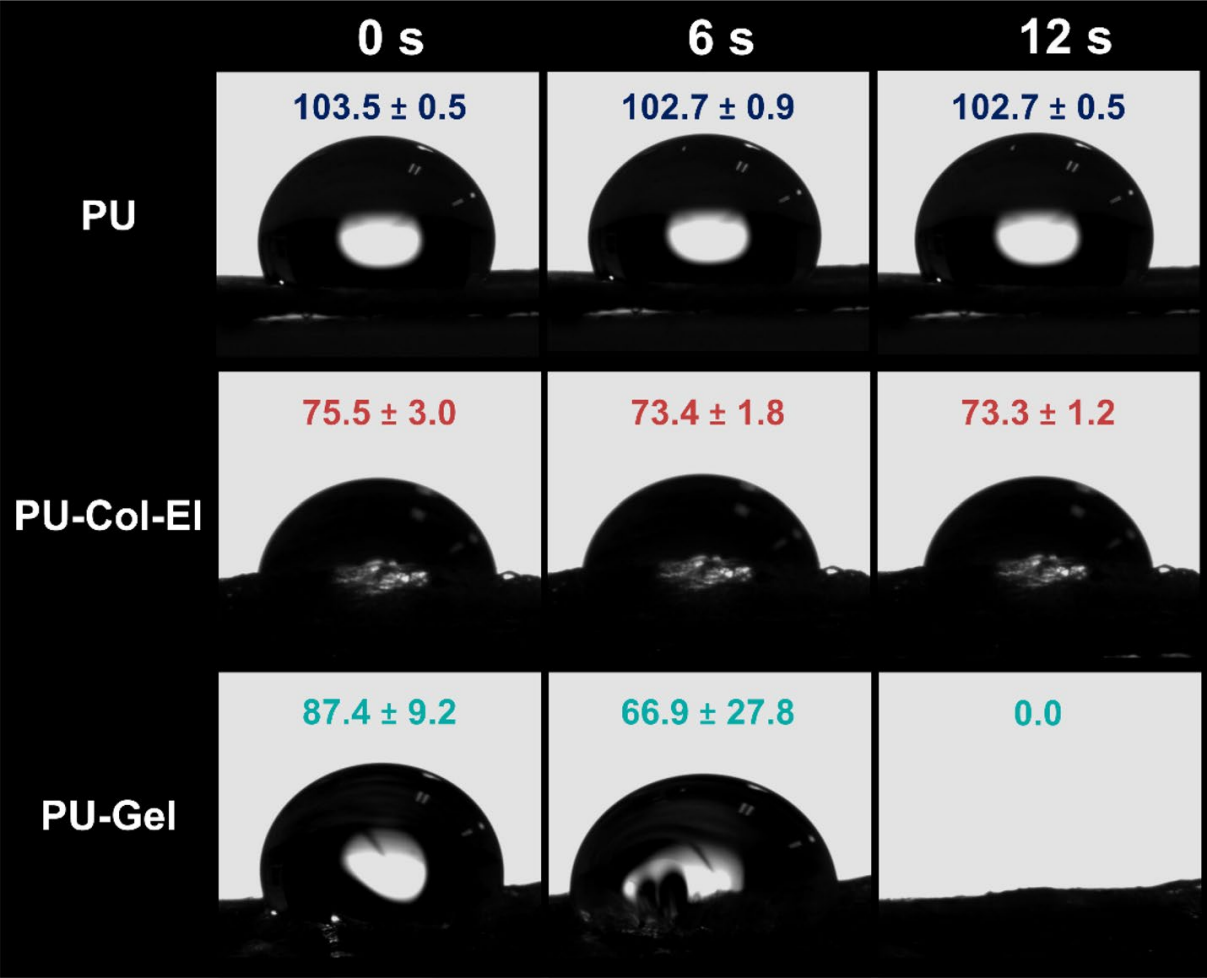
Dynamic contact angle measurements for the scaffolds.

Dynamic contact angle (CA) measurements were used to assess the wettability of each scaffold. Dynamic CA is a more reliable metric than static CA for wettability evaluation, as it denotes CA differences over time (Fig. 4). CAs were greater than 90° for PU scaffolds for 12 s, which is in line with their hydrophobicity. PU-Col-El scaffolds yielded steady CA values (lower than 90°) indicating the hydrophilicity of the scaffolds. CA values for PU-Gel scaffolds also decreased to 0° within 12 s, indicating strong hydrophilicity.

**Figure 5:**
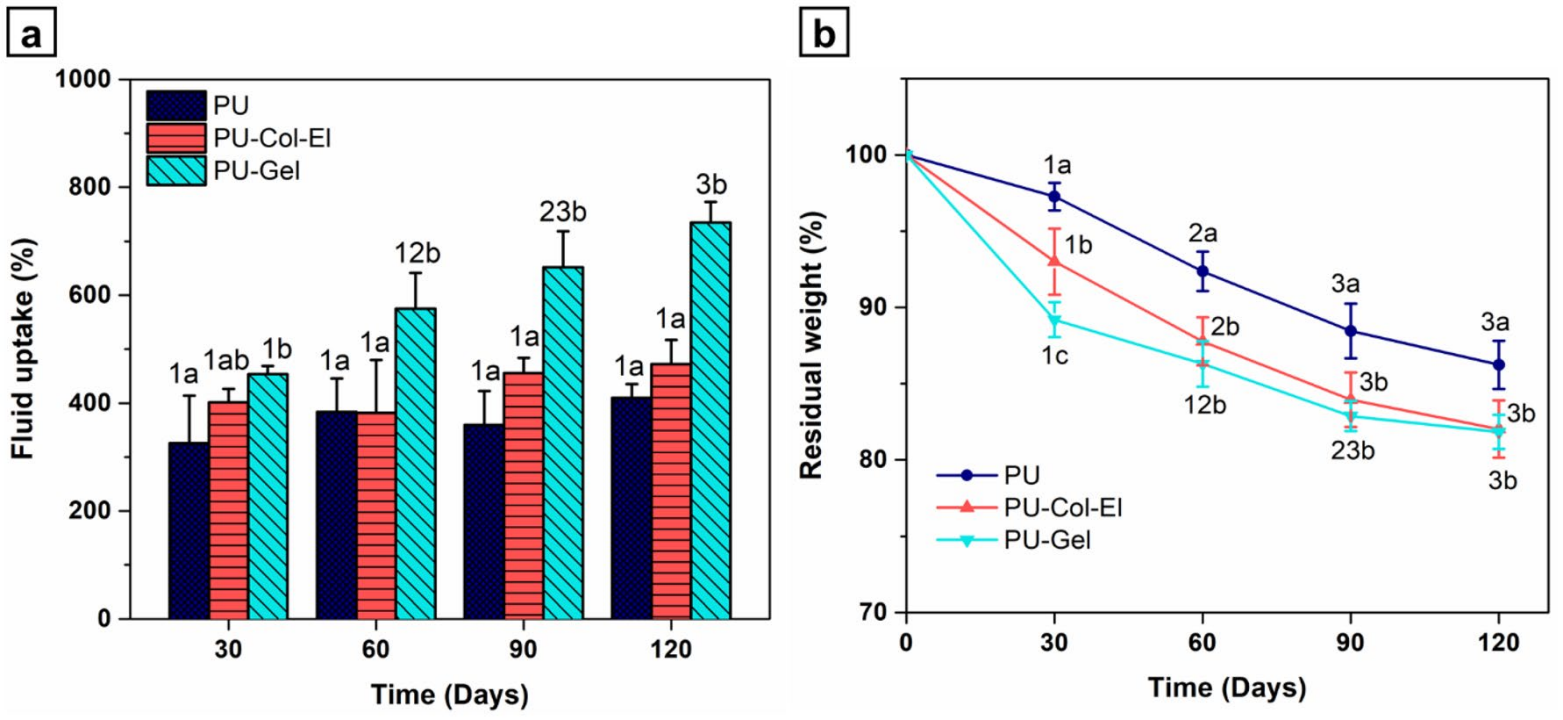
Time profiles of (**a**) fluid uptake and (**b**) in vitro degradation of scaffolds. Statistical differences were analyzed using two-way ANOVA with a Bonferroni post-hoc test (a, b, c denotes a significant difference of p < 0.05 between scaffolds; and 1, 2, 3 indicates a significant difference of p < 0.05 between time points).

The fluid uptake levels of the scaffolds were also evaluated using the phosphate-buffered saline (PBS) in vitro swelling test. All scaffolds showed high fluid uptake from 30 to 120 days of cultivation (Fig. 5a), which is intrinsic to the porous nature of the scaffolds^21^. Protein addition increased the swelling ability of the synthetic polymer, which is in line with previous findings^22,23^. The PU-Gel scaffold had the highest fluid uptake (734.6% ± 38.1% in 120 days), with a statistically significant difference (p < 0.05) from that of the PU scaffold across all time points. This confirms the hydrophilicity of protein-blended PU scaffolds, is in line with dynamic CA measurements.

Scaffolds were subjected to in vitro degradation tests using PBS to assess hydrolytic degradation over time (Fig. 5b). PU-Col-El and PU-Gel scaffolds yielded higher degradation rates than that of PU at all time points. At 120 days, PU-Col-El and PU-Gel decreased in weight by 18.0% ± 1.9% and 18.2% ± 1.1%, respectively. The residual weights obtained are consistent with previous findings on synthetic and natural polymer blends^24,25^.

**Figure 6:**
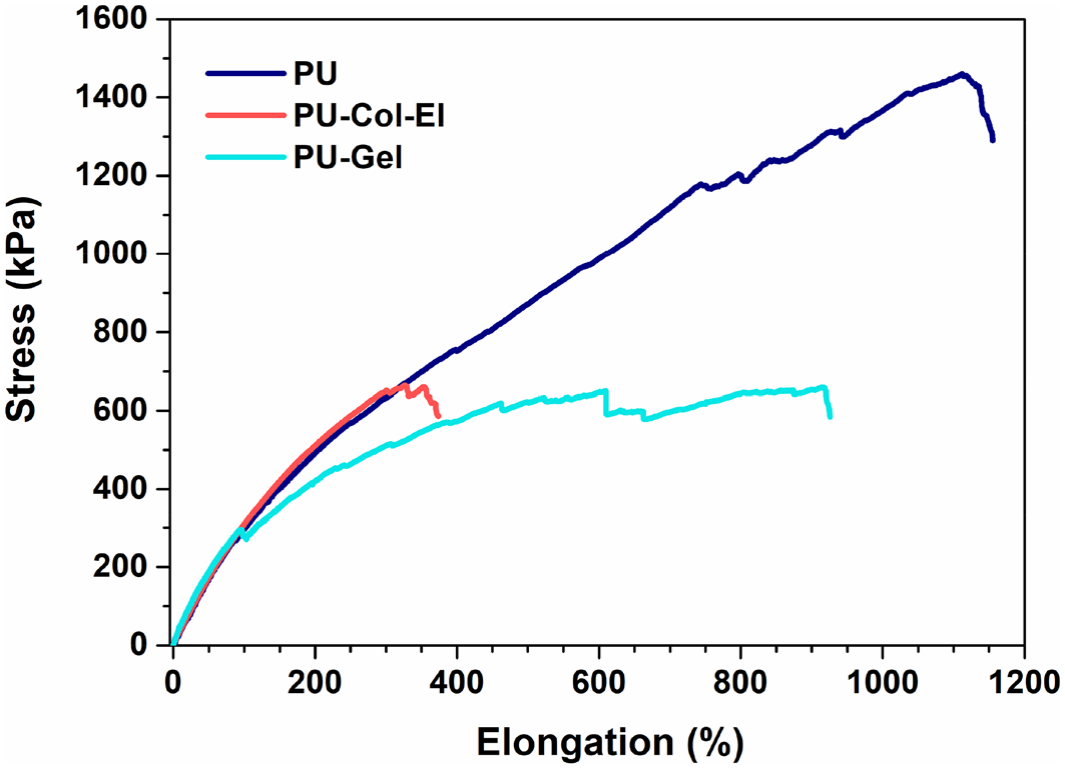
Representative stress vs. elongation curves of PU, PU-Col-El, and PU-Gel scaffolds.

A uniaxial tensile test was performed to examine the mechanical strength, elongation at break, and stiffness of the scaffolds. Figure 6 shows representative stress–strain curves, and averages of the mechanical property measurements were obtained from the stress–strain curves (see Supplementary Table S1 online). The addition of proteins to the PU scaffold significantly (p < 0.05) reduced the mechanical strength and elongation at break in the PU-Col-El and PU-Gel scaffolds compared to that in the PU scaffold. Similar results with protein addition to synthetic polymers have been previously observed^26,27^. On the other hand, the stiffness of the scaffolds remained unaffected (p < 0.05) upon the addition of Col and El or Gel to the PU. The PU-Gel scaffold was more ductile than the PU-Col-El scaffold.

### Bioactivity of scaffolds for vascular graft applications

**Figure 7:**
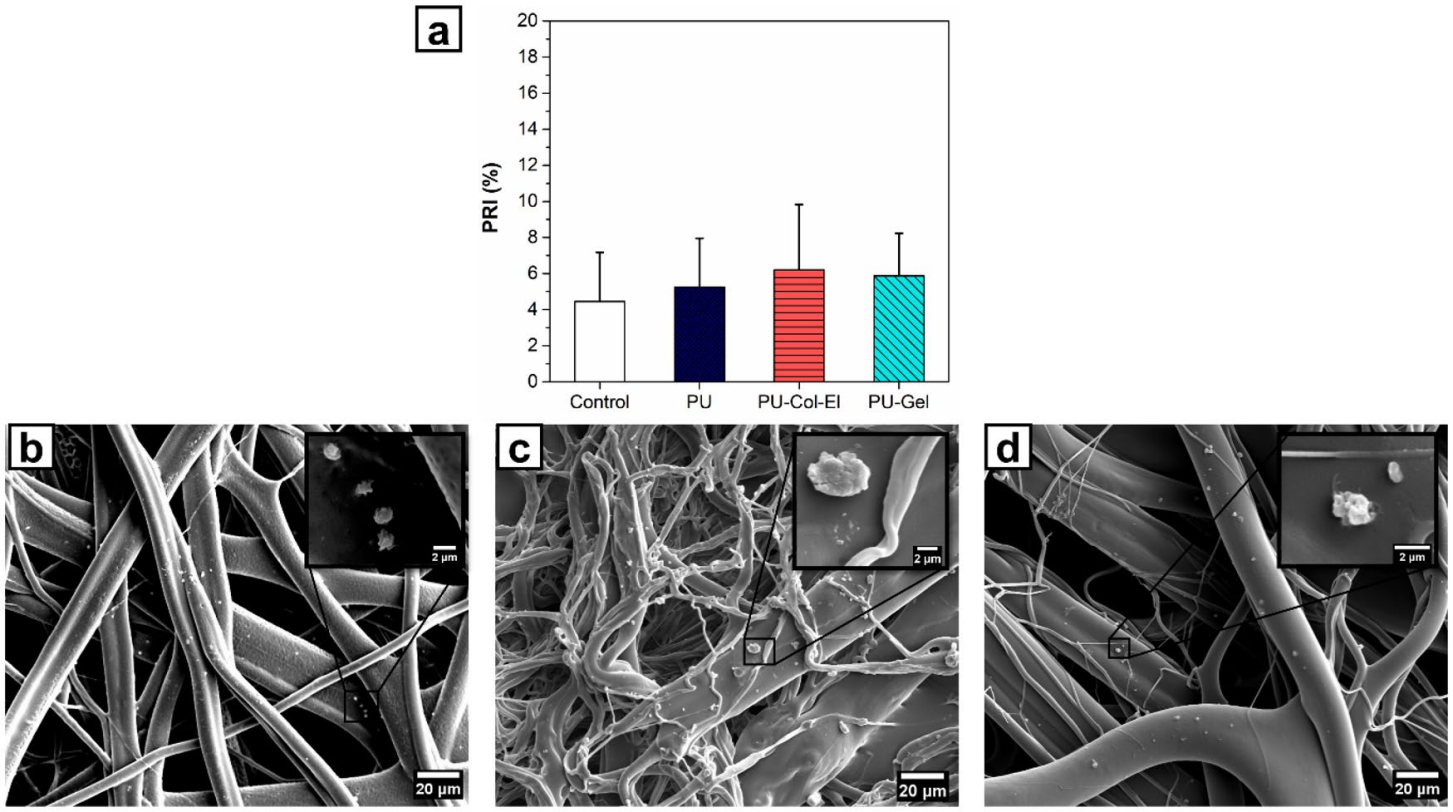
(**a**) Platelet retention index (PRI) of the scaffolds, and platelet adhesion onto (**b**) PU, (**c**) PU-Col-El, and (**d**) PU-Gel scaffolds.

Platelet adhesion on the scaffolds was quantified based on the platelet retention index (PRI) values obtained over 90 min (Fig. 7a). The PRI values obtained for PU, PU-Col-El, and PU-Gel were 5.5% ± 1.2%, 6.7% ± 2.0%, and 7.7% ± 1.7%, respectively. There was no significant difference (p > 0.05) in platelet adhesion on scaffolds compared to that in the control (4.8% ± 1.7%).

We next evaluated the morphology of adhered platelets on the scaffolds to assess platelet activation. Figure 7b–d shows SEM images of platelets interacting with the scaffolds. Platelets have different morphologies according to their activation stages^28^. Platelet activation can be determined by observing the level of agglomeration and hyaloplasm spreading on the surface^29^. In contrast, rounded and distributed platelets are inactivated^30^. Images shown in Fig. 7b–d thus indicates that the platelets adhering onto the PU, PU-Col-El, and PU-Gel scaffolds did not show an activation profile.

**Figure 8:**
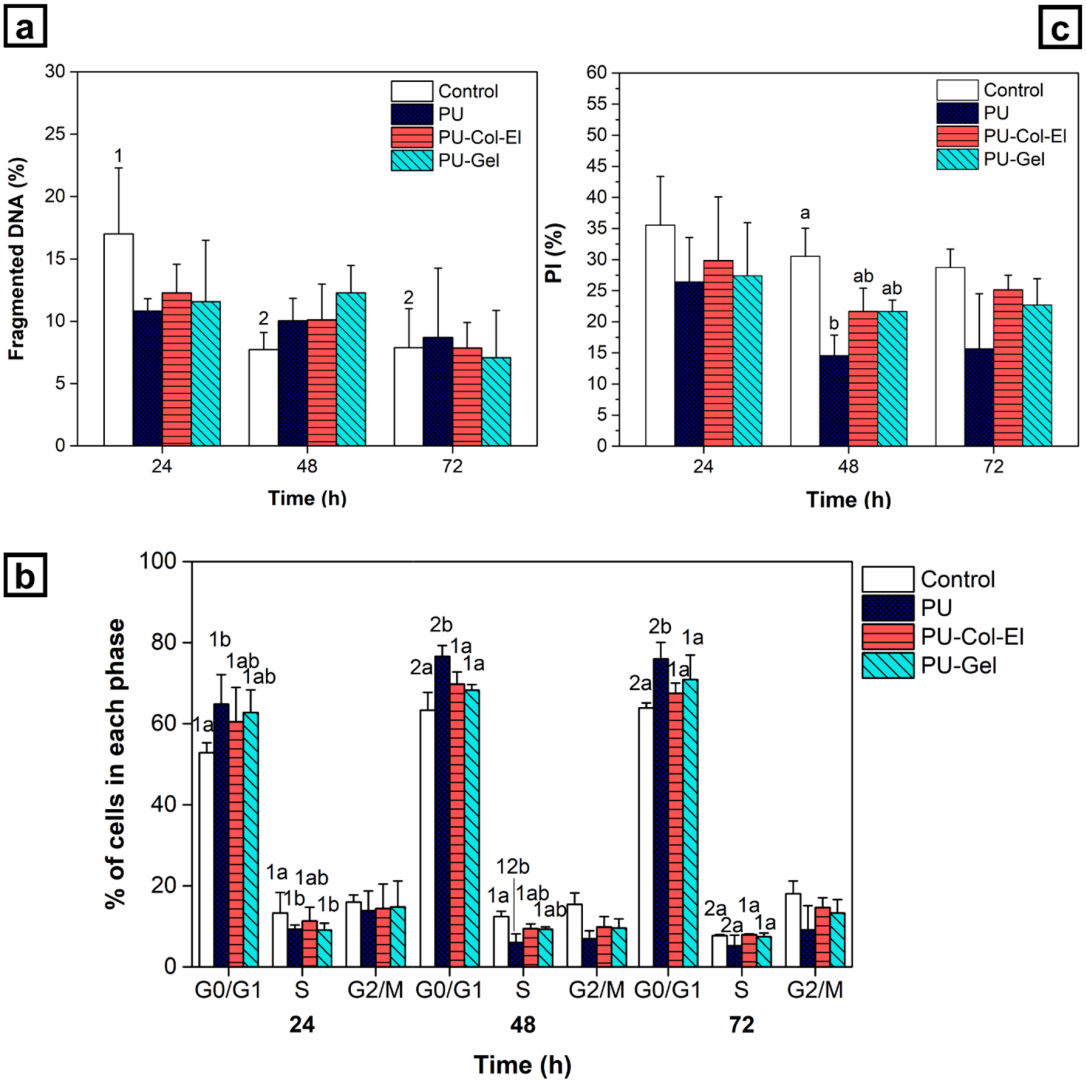
(**a**) Fragmented DNA, (**b**) cell cycle profile, and (**c**) proliferation index (PI) determined using flow cytometry in HUVECs treated with scaffolds. Statistical differences were analyzed using two-way ANOVA with a Bonferroni post-hoc test (a, b, c denotes a significant difference of p < 0.05 between scaffolds, while 1, 2, 3 indicates a significant difference of p < 0.05 between time points).

For successful vascular applications, the contact between the scaffolds and endothelial cells must not compromise cell viability and proliferation. Cell viability analysis of scaffold production methods involving the use of solvents, such as RJS, is therefore crucial to ensure no cytotoxic effects are present^31^. We thus analyzed the cell cycle of endothelial cells using flow cytometry to investigate cellular proliferation and apoptosis profiles. The percentage of cells with fragmented DNA was used to represent the proportion of apoptotic cells (Fig. 8a). Within the time period studied (24, 48, and 72 h), no significant differences (p > 0.05) in terms of apoptosis were found for any of the scaffolds compared to the control group.

Figure 8b shows the percentage of cells in each phase after 24, 48, and 72 h. The PU scaffold showed delayed cell proliferation, with an increase in the number of cells in G0/G1 phase and a decrease in those in S and G2/M phases. Conversely, the PU-Col-El scaffold had no differences compared to the control group and the PU-Gel scaffold at all time points and cell cycle phases. The PU scaffold also showed the lowest cell proliferation according to the proliferation index (Fig. 8c).

**Figure 9:**
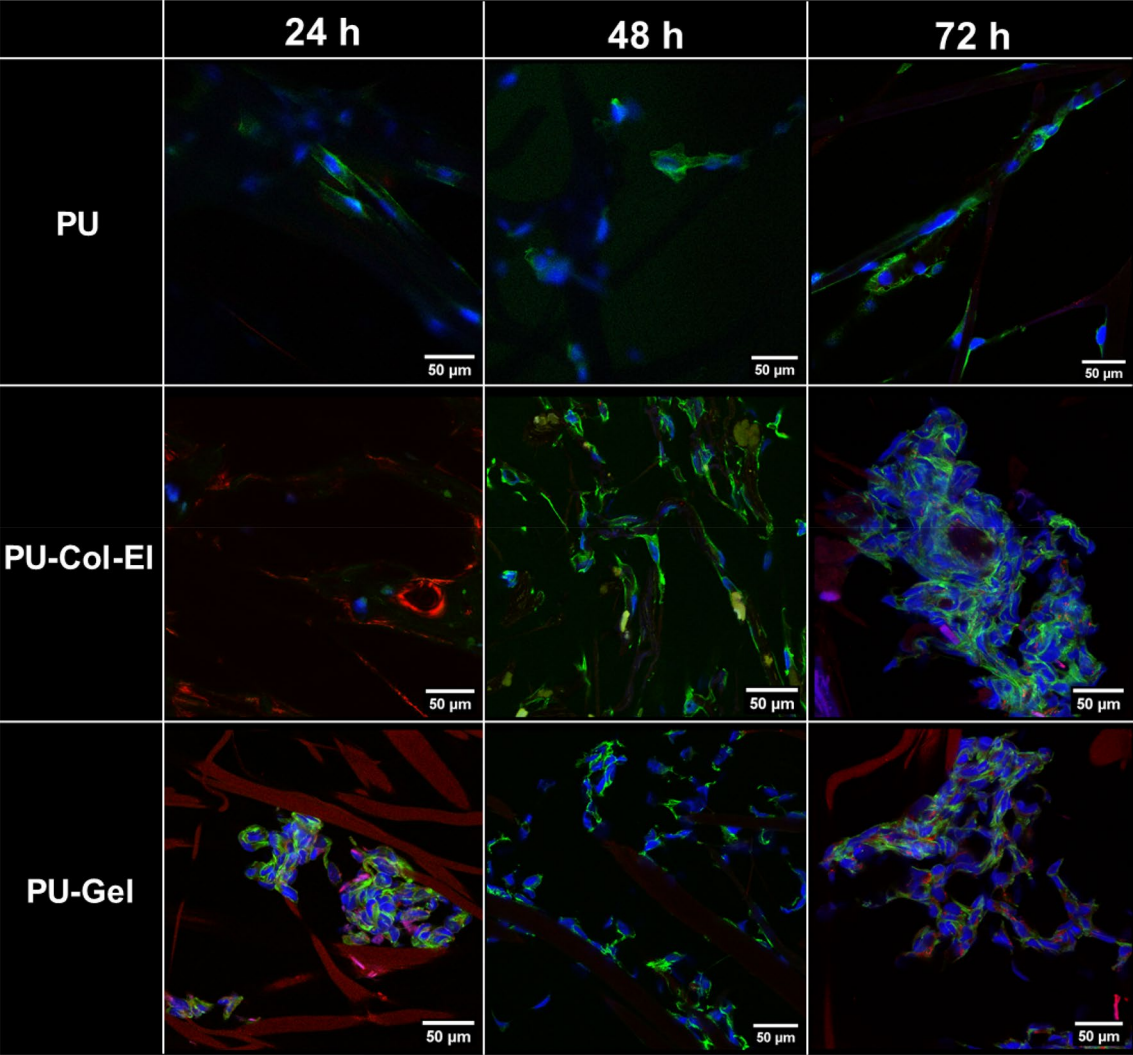
Fluorescent images of HUVECs on PU, PU-Col-El, and PU-Gel scaffolds obtained using confocal microscopy. The green color corresponds to the cytoplasm, blue to the cell nuclei, and red to the scaffold fibers (on PU-Col-El and PU-Gel scaffolds).

Finally, confocal microscopy was used to qualitatively analyze HUVEC morphology on the scaffolds (Fig. 9). There was a remarkable difference in cell morphology between the scaffolds. Elongated cell shapes were observed along the fibers of the PU scaffolds. On the other hand, cells on PU-Col-El scaffolds were more aggregated, and tended to initiate monolayer formation, which is in line with the typical physiological features of HUVECs. Cells on PU-Gel scaffolds presented both of these morphologies, with a tendency to aggregate over time. The supplementary video attached to the electronic version of the present study shows a more detailed 3D visualization of the scaffolds and cells (see Supplementary Video 4–6 online).

## Discussion

In the present study, we demonstrated the successful combination of several ECM proteins with PU to produce scaffolds for vascular graft applications. Fibrous scaffolds with homogenous and micrometric fiber diameters were produced using the RJS method, and verified via SEM analysis. The fiber diameters of the PU-Col-El scaffolds were narrower than those of the PU and PU-Gel scaffolds. Fiber diameter affects the endothelial cell response^32^ and mechanical properties^33^ of scaffolds. Porosity is also an important factor to support vascularization, nutrients and cell diffusion throughout the scaffolds^34,35^. The 3D microstructure of PU, PU-Col-El and PU-Gel scaffolds was porous and interconnected, and there was no significant change in porosity after protein addition. Protein-based fibers usually display uniform and smooth surfaces^36^. However, a certain degree of nanometric surface roughness is also advantageous for tissue engineering applications. In the present study, nanometric roughness of the PU matrix was observed for both PU-Col-El and PU-Gel fibers. These features should be beneficial for vascular grafting, as this leads to improved cell differentiation^37^, adhesion, and proliferation profiles^38,39^.

The contact angles of the scaffolds indicated that the PU-Gel was the most hydrophilic of the tested scaffolds. The higher hydrophilicity of Gel-containing scaffolds compared to Col and El may be due to two factors. First, the Gel deposits on the fiber surface observed using AFM are hydrophilic. Second, Col and El have more hydrophobic structures than Gel (i.e., hydrolyzed collagen)^40^. With the increased hydrophilicity due to the addition of proteins, especially gelatin, the fluid uptake ability of the PU scaffolds increased. The in vitro swelling of scaffolds corresponds to the fluid uptake of the cell culture medium or body fluids^41^. Swelling thus has a crucial impact on scaffold exposure to hydrolysis, nutrient transport, cell adhesion, proliferation, and differentiation^41,42^.

The hydrolytic degradation profile of the scaffolds is another important property, as it affects tissue regeneration and the structural integrity of scaffolds^25^. The total protein degradation time in scaffolds varies from twelve hours to five months^9^. In the present study, the addition of ECM proteins increased the degradation rate of PU. The higher degradation rates may be attributed to the intermolecular bonds formed between PU and the proteins within the scaffold^31^. Under ideal conditions, the degradation rate must be such that scaffold integrity is maintained during graft surgery, while allowing new tissue formation^43^. The growth rate of new vascular tissue is still unknown, and it depends on several physical, chemical, and biological factors associated with the physiology and tissue interactions with the scaffold^4^. We observed an increase in weight loss over time, which was directly related to the addition of ECM proteins into the PU. Beyond hydrolytic degradation, scaffold polymers are also degraded via other mechanisms within the human body, such as oxidative and enzymatic degradation. Degradation via these additional mechanisms should be explored thoroughly in future studies.

The mechanical properties of grafts are essential parameters that can establish their suitability for vascular applications. Scaffolds must match the elasticity and strength of the native tissue to ensure optimal integration with the body under physiological conditions (for example, blood pressure). Tensile tests are used to assess the strength, elongation, and stiffness of grafts^44^. All the scaffolds in the present study withstood large elongation, and their stiffness levels did not change significantly (p > 0.05) with the addition of ECM proteins to PU (Table S1). Although the addition of ECM proteins reduced the tensile strength of the scaffolds significantly (p < 0.05), this may not be a problem as tensile strengths as low as 100 kPa have been reported previously for blood vessels^45^. Nevertheless, as tensile strength is directly proportional to burst pressure^44^, methods to improve the mechanical properties of such scaffolds should be investigated. To this end, crosslinking the scaffolds with proteins^30^ and growth factors to increase ECM production in cells may be a promising avenue. In addition, other functional mechanical properties, such as suture retention strength and compliance, should also be evaluated in the future.

The occurrence of initial hemostatic events within the vascular grafts, including platelet adhesion and activation, should be carefully evaluated, as these events are directly related to the occurrence of thrombosis and implant failure^12^. In the present study, the tested scaffolds did not induce platelet adhesion and activation. Although the presence of Col within the scaffold may promote platelet adhesion, it may not be deposited on the fiber surface of the scaffold, and the addition of El can reduce this thrombogenic effect^46^. Furthermore, although Gel may be deposited on the fiber surface, the high hydrophilicity of the PU-Gel scaffolds may also be associated with the absence of significant (p > 0.05) platelet adhesion^12^. These results suggest that the scaffolds produced here are unlikely to cause thrombosis or lead to vascular failure.

Flow cytometry analysis indicated good cytocompatibility of scaffolds with endothelial cells, since the scaffolds did not trigger apoptosis and were not cytotoxic. The blended scaffolds (PU-Gel and PU-Col-El) had better cytocompatibility than the PU scaffold. A reduction in cell proliferation compared to that of the control was also observed for the PU scaffolds. The MTT assay results in our previous studies^17,18^ agree with these findings, confirming that HUVEC viability significantly decreased (p > 0.05) after 48 h compared to that of the control, PU-Col-El, and PU-Gel scaffolds (Supplementary Fig. S1 online). Based on previous studies, the addition of Col and El is expected to yield the best cytocompatibility profile^22,26^, yet cell viability and proliferation with the PU scaffold also increased upon the addition of Gel, and were not significantly different (p < 0.05) to those of PU-Col-El.

Differences in cell morphology were also observed between the PU, PU-Col-El, and PU-Gel scaffolds. HUVECs were elongated along the PU fibers, while aggregated on the PU-Col-El and PU-Gel scaffolds over time. The elongation of cells is expected to occur more on PU scaffolds because of the larger fiber diameters and roughness compared to PU-Col-El and PU-Gel scaffolds. Fioretta et al*.* previously demonstrated that HUVEC morphology varies according to distinct fiber diameters, and tends to elongate in fibers that are slightly smaller than cell size^32^. In contrast, the aggregation of HUVECs on PU-Col-El and PU-Gel may be related to the interaction of these cells with the ECM proteins. Cell aggregation is propitious, as it indicates a possible path to the physiological confluence of endothelial cells for endothelium formation^47^.

In summary, protein addition to PU increased the hydrophilicity, thus increasing hydrolytic degradation of the scaffolds. It also improved cell viability, adhesion, and proliferation, and did not induce platelet adhesion on scaffolds. The PU-Col-El and PU-Gel scaffolds supported better viability and proliferation of HUVECs than the PU scaffold. Adhesion and aggregation of HUVECs was observed on both the PU-Col-El and PU-Gel scaffolds, suggesting endothelial formation. PU-Gel scaffolds presented similar interactions with HUVECs, with no substantial differences compared to PU-Col-El scaffolds, while the ductility of PU-Gel was considerably higher than that of PU-Col-El. However, the cost of raw materials to produce PU-Col-El scaffolds is approximately 100 times higher than that of PU-Gel scaffolds. Accordingly, PU-Gel was the most promising scaffold for vascular applications, considering the statistically equivalent results with those of PU-Col-El scaffolds with respect to interaction with HUVECs and higher ductility, yet lower cost. Thus, the combination of proteins and vascular graft scaffolds is a plausible and attractive alternative to produce materials with improved compatibility for vascular tissue engineering, but the choice of graft material should still take the manufacturing costs into account, while considering the specific demands.

## Materials and methods

### Rotary jet spinning of scaffolds

Type I collagen (C9879), type B gelatin (G9391), elastin (E1625), and 1,1,1,3,3,3‐hexafluoro‐2‐propanol (HFIP) were purchased from Sigma-Aldrich. Chloroform was purchased from LabSynth (São Paulo, Brazil). Polyurethane (Tecoflex, SG85A) was obtained from Lubrizol Advanced Materials (Wickliffe, OH, USA). PU, PU-Col-El, and PU-Gel solutions were prepared at a total concentration of 9% (w/v) polymers in solvent. The preparation of the PU solution in chloroform and RJS process to obtain scaffolds with random fibers were performed according to a method described previously^19^. The blended solutions of PU and proteins were mixed at a ratio of 75/25 w/w in HFIP for both PU-Col-El and PU-Gel. The relative ratio of Col/El was 75/25. The rotary jet-spun scaffolds of PU and PU-Gel were produced at 6000 rpm, while PU-Col-El scaffolds were produced at 18,000 rpm. The fibers were collected at 17 cm from the reservoir. The equipment used for this process was described in detail in a previous study^19^.

### Scaffold morphology and surface characterization

The scaffold morphology was characterized via scanning electron microscopy (SEM) using a ZEISS (Oberkochen, Germany) EVO MA 15 microscope operated at an accelerated voltage of 10 kV. Samples were gold-sputtered (Quorum SC7620) before SEM imaging. The fiber diameter distribution was obtained by measuring 50 fibers using ImageJ software (ImageJ, National Institutes of Health). Atomic force microscopy (AFM) in the intermittent-contact mode was used to characterize the fiber surface with an NX10 Parksystems microscope. Cantilevers were used with a resonance frequency of 75 kHz and a force constant of 2.8 N/m. Images were obtained on a 5 × 5 μm surface area, and expressed as height and phase images. Second-order polynomial leveling was applied to the height models to determine the average roughness (Ra) using Gwyddion software version 2.54.

### Porosity characterization

The scaffold porosity was assessed using gravimetric analysis and microcomputed tomography (Micro-CT). Gravimetry was performed using ethanol for liquid intrusion^48,49^. Briefly, the scaffold area (A) and thickness (E) of three samples was measured using a micrometer. The dry weight of samples (W_d_) was recorded and samples were immersed overnight in ethanol (ρ = 0.79 g/cm^3^) to obtain the wet weight (W_e_). The scaffold porosity was calculated using Eq. (1).

1 $$\mathrm{Porosity }\,\left(\mathrm{\%}\right)=\frac{{W}_{e}-{W}_{d}}{\rho *E*A}*100$$

The microstructure and porosity were also characterized using micro-CT (Metrotom 800, ZEISS, Oberkochen, Germany). The samples were scanned using 13 µm of voxel resolution, 1000 ms per frame of integration time, and a total of 800 projections at a 40 kV voltage and 200 μA current. 2D sliced images of the tomograms were segmented and the percent porosity was measured using the DiameterJ plugin of ImageJ^50^. A 3D image was generated from the 2D slice image compilation.

### Determination of wettability

The dynamic contact angle was used to measure the wettability of the scaffolds, and was determined using the sessile drop technique with an optical contact angle device (OCA15, Dataphysics, San Jose, CA, USA). A sessile distilled water drop was deposited onto the surface of the scaffold using a syringe, and the drop contour was measured and fitted using the Dataphysics SCA20 software. Three samples were used to measure the contact angles for 12 s.

### Determination of hydrolytic degradation and fluid uptake

RJS-scaffolds were dried to a constant weight (W_0_), and immersed in 3 mL of phosphate-buffered saline (PBS, at 0.1 M and pH 7.4). Three samples of each scaffold were incubated at static conditions at 37 °C for each degradation time point (30, 60, 90, and 120 days). After incubation, excess PBS was removed, and the weight was recorded (W_1t_) to calculate fluid uptake (Eq. 2). Subsequently, hydrolytic degradation was calculated according to the ASTM F1635-11 standard. The samples were washed with distilled water, and dried to a constant weight (W_2t_) to calculate the residual mass as follows (Eq. 3):

2 $$\mathrm{Fluid \,uptake }\left(\mathrm{\%}\right)=\frac{{W}_{1\mathrm{t}}-{W}_{2\mathrm{t}}}{{W}_{2\mathrm{t}}}*100$$

3 $$\mathrm{Residual\, weight }\,\left(\mathrm{\%}\right)=\frac{{\mathrm{W}}_{2\mathrm{t}}}{{W}_{0}}*100$$

### Determination of scaffold mechanical properties

Tensile tests were performed according to the ASTM D882-12 standard. The scaffolds were first cut into 60 × 15 mm rectangular pieces, conditioned at 20 °C, and transferred to pneumatic grips (Biopuls, Instron) of 25 × 25 mm. The tensile properties were measured based on the apparent area of the scaffolds at a crosshead speed of 5 mm/min using a uniaxial load test machine (Instron) equipped with a 10 N load cell, and controlled using Bluehill Universal software (Instron). Stress–strain curves were generated to visualize the mechanical strength, stiffness, and elongation at break of the scaffolds. Statistical results were considering the average of five samples, and a representative sample was used as a reference.

### Determination of platelet adhesion and activation

Scaffolds were sterilized using ultraviolet (UV) light before being placed in 24-well plates and equilibrated in PBS for 2 h at 37 °C. The PBS was removed, and the scaffolds and control group (without scaffold) were incubated with 600 μL of platelet-rich plasma (PRP) at a concentration of 1.3 × 10^6^ platelets µL^−1^ for 90 min at 37 °C in a rotary shaker at 200 rpm. PRP was obtained from human blood collected from healthy blood donors. All experiments were performed in accordance with the relevant guidelines and regulations, and informed consent was obtained from all participants and/or their legal guardians. The ethical committee of the University of Campinas (CAAE: 26437519.8.0000.5404) approved the use of human blood for this study. The PRP was removed, the wells were rinsed with PBS, the supernatant was collected, and the platelets were counted immediately using a hemocytometer. Quantitative analysis of platelet adhesion to scaffolds was performed using the Salzman’s platelet retention index (PRI) (Eq. 4), where PLTi and PLTe are the platelet concentrations before and after adhesion, respectively. The statistical results are presented as the average of nine independent samples.

4 $$\mathrm{PRI }\,\left(\mathrm{\%}\right)=\frac{{\mathrm{PLT}}_{\mathrm{i}}-{\mathrm{PLT}}_{\mathrm{e}}}{{\mathrm{PLT}}_{\mathrm{i}}}*100$$

Adhered platelets on the scaffolds were fixed with 2.5% (v/v) glutaraldehyde in PBS for 30 min. Each sample was washed twice with PBS for 10 min, and dehydrated using an ethanol gradient (30, 50, 70, 90, and 100% ethanol for 15 min each). Finally, the scaffolds were dried in a vacuum desiccator for 12 h, gold-sputtered, and observed using SEM.

### Cell culture and cell cycle determination using flow cytometry

Dulbecco’s modified Eagle’s medium (DMEM) was supplemented with 2 mM L-glutamine, 10% fetal bovine serum, 100 μg mL^−1^ streptomycin, and 100 U mL^−1^ penicillin (Life Technologies Inc., USA). Human umbilical vein endothelial cells (HUVECs) were cultured from passages three and four in DMEM under 5% CO_2_ at 37 °C.

For the flow cytometry assay, HUVECs were seeded in 24-well plates at a density of 5.5 × 10^4^ per well in 300 μL of culture medium for 24 h under 5% CO_2_ and 37 °C. Scaffolds were cut into 15 mm diameter discs, sterilized overnight by UV light, and added to the wells. The cells treated with scaffolds and the control group (untreated) were incubated for 24, 48, and 72 h. Subsequently, cells were harvested, washed with PBS, fixed with cold ethanol solution (70%) for 30 min, and incubated with 50 μg mL^−1^ of propidium iodide in PBS containing 1 mg mL^−1^ of RNase at room temperature in the dark for 30 min. Flow cytometry analysis was performed to measure the percentage of cells in different cell cycle phases using Accuri ™ C6 software (BD Biosciences). The experiments were carried with three samples each and repeated three times. The proliferation index (PI) was calculated using the following equation:

5 $$\mathrm{PI }\,\left(\mathrm{\%}\right)=\frac{\mathrm{S}+\mathrm{G}2/\mathrm{M}}{\mathrm{G}0/\mathrm{G}1+\mathrm{S}+\mathrm{G}2/\mathrm{M}}*100$$

### Determination of cell morphology and adhesion

Scaffolds sterilized with UV light overnight were used to seed HUVECs at a density of 1.0 × 10^5^ per well in 400 μL of culture medium and were then incubated under 5% CO_2_ and 37 °C for 24, 48, and 72 h. At each time point, the culture medium was discarded, and the cells were washed with PBS and fixed with 3.7% (v/v) formaldehyde for 5 min. The scaffolds were then washed with PBS, and the cells were stained using 50 μg mL^−1^ phalloidin (cytoplasm) for 40 min at room temperature. The scaffolds were washed with PBS, stained with 0.1 μg mL^−1^ of DAPI (nuclei) for 15 min at 37 °C, and washed again with PBS. Fluorescence images were obtained using a confocal microscope (Leica TCS SP5 II).

### Statistical analysis

Scaffolds and different time points were compared using one-way ANOVA or two-way ANOVA, followed by a post-hoc Bonferroni test. Statistical analysis was carried out using IBM SPSS Statistics 22.0 software. Data was tested for normality using the Shapiro–Wilk test. The results are presented as the mean ± standard deviation (SD). Values of p < 0.05 were considered to be statistically significant.

## Supplementary Information

Supplementary Information 1.

Supplementary Video 1.

Supplementary Video 2.

Supplementary Video 3.

Supplementary Video 4.

Supplementary Video 5.

Supplementary Video 6.

## References

[1] Abbafati C (2020). Global burden of 369 diseases and injuries in 204 countries and territories, 1990–2019: A systematic analysis for the Global Burden of Disease Study 2019. The Lancet.

[2] Copes F, Pien N, Van Vlierberghe S, Boccafoschi F, Mantovani D (2019). Collagen-based tissue engineering strategies for vascular medicine. Front. Bioeng. Biotechnol..

[3] Wang Z, Mithieux SM, Weiss AS (2019). Fabrication techniques for vascular and vascularized tissue engineering. Adv. Healthcare Mater..

[4] Li J, Chen Z, Yang X (2019). State of the art of small-diameter vessel-polyurethane substitutes. Macromol. Biosci..

[5] Coenen AMJ, Bernaerts KV, Harings JAW, Jockenhoevel S, Ghazanfari S (2018). Elastic materials for tissue engineering applications: Natural, synthetic, and hybrid polymers. Acta Biomater..

[6] Sorushanova A (2019). The collagen suprafamily: From biosynthesis to advanced biomaterial development. Adv. Mater..

[7] Fu W (2014). Electrospun gelatin/PCL and collagen/PLCL scaffolds for vascular tissue engineering. Int. J. Nanomed..

[8] Elsayed Y, Lekakou C, Labeed F, Tomlins P (2016). Fabrication and characterisation of biomimetic, electrospun gelatin fibre scaffolds for tunica media-equivalent, tissue engineered vascular grafts. Mater. Sci. Eng. C.

[9] Goonoo N, Bhaw-Luximon A, Bowlin GL, Jhurry D (2013). An assessment of biopolymer- and synthetic polymer-based scaffolds for bone and vascular tissue engineering. Polym. Int..

[10] Jana S (2019). Endothelialization of cardiovascular devices. Acta Biomater..

[11] Liliensiek SJ (2010). Modulation of human vascular endothelial cell behaviors by nanotopographic cues. Biomaterials.

[12] Vatankhah E (2014). Electrospun tecophilic/gelatin nanofibers with potential for small diameter blood vessel tissue engineering. Biopolymers.

[13] Kitsara M, Agbulut O, Kontziampasis D, Chen Y, Menasché P (2017). Fibers for hearts: A critical review on electrospinning for cardiac tissue engineering. Acta Biomater..

[14] Khang A (2017). Engineering anisotropic biphasic Janus-type polymer nanofiber scaffold networks via centrifugal jet spinning. J. Biomed. Mater. Res. B Appl. Biomater..

[15] Zeugolis DI (2008). Electro-spinning of pure collagen nano-fibres—Just an expensive way to make gelatin?. Biomaterials.

[16] Freedman KJ, Haq SR, Edel JB, Jemth P, Kim MJ (2013). Single molecule unfolding and stretching of protein domains inside a solid-state nanopore by electric field. Sci. Rep..

[17] Rodrigues ICP (2020). A novel technique to produce tubular scaffolds based on collagen and elastin. Artif. Organs.

[18] Rodrigues ICP (2020). Low-cost hybrid scaffolds based on polyurethane and gelatin. J. Market. Res..

[19] Rodrigues ICP (2020). Polyurethane fibrous membranes tailored by rotary jet spinning for tissue engineering applications. J. Appl. Polym. Sci..

[20] Ho ST, Hutmacher DW (2006). A comparison of micro CT with other techniques used in the characterization of scaffolds. Biomaterials.

[21] Grover CN, Cameron RE, Best SM (2012). Investigating the morphological, mechanical and degradation properties of scaffolds comprising collagen, gelatin and elastin for use in soft tissue engineering. J. Mech. Behav. Biomed. Mater..

[22] Aguirre-Chagala YE (2017). Physicochemical properties of polycaprolactone/collagen/elastin nanofibers fabricated by electrospinning. Mater. Sci. Eng. C.

[23] Gong Y, Zhou Q, Gao C, Shen J (2007). In vitro and in vivo degradability and cytocompatibility of poly(l-lactic acid) scaffold fabricated by a gelatin particle leaching method. Acta Biomater..

[24] Chan-Chan LH (2013). Characterization and biocompatibility studies of new degradable poly(urea)urethanes prepared with arginine, glycine or aspartic acid as chain extenders. J. Mater. Sci. Mater. Med..

[25] Xiang P (2018). The in vitro and in vivo biocompatibility evaluation of electrospun recombinant spider silk protein/PCL/gelatin for small caliber vascular tissue engineering scaffolds. Colloids Surf. B.

[26] Wong CS, Liu X, Xu ZG, Lin T, Wang XG (2013). Elastin and collagen enhances electrospun aligned polyurethane as scaffolds for vascular graft. J. Mater. Sci. Mater. Med..

[27] Strobel HA, Calamari EL, Beliveau A, Jain A, Rolle MW (2018). Fabrication and characterization of electrospun polycaprolactone and gelatin composite cuffs for tissue engineered blood vessels. J. Biomed. Mater. Res. B Appl. Biomater..

[28] Farhatnia Y (2016). Next generation covered stents made from nanocomposite materials: A complete assessment of uniformity, integrity and biomechanical properties. Nanomed. Nanotechnol. Biol. Med..

[29] Jing X (2015). Electrospinning thermoplastic polyurethane/graphene oxide scaffolds for small diameter vascular graft applications. Mater. Sci. Eng. C.

[30] Dong H (2018). Enhanced performance of magnesium alloy for drug-eluting vascular scaffold application. Appl. Surf. Sci..

[31] Heiny M, Shastri VP (2016). Nanofibers of elastin and hydrophilic segmented polyurethane solution blends show enhanced mechanical properties through intermolecular protein-polymer H bonding. Biomacromol.

[32] Fioretta ES, Simonet M, Smits AIPM, Baaijens FPT, Bouten CVC (2014). Differential response of endothelial and endothelial colony forming cells on electrospun scaffolds with distinct microfiber diameters. Biomacromol.

[33] Kim HH, Kim MJ, Ryu SJ, Ki CS, Park YH (2016). Effect of fiber diameter on surface morphology, mechanical property, and cell behavior of electrospun poly(ε-caprolactone) Mat. Fibers Polym..

[34] Sharifpoor S, Simmons CA, Labow RS, Santerre JP (2010). A study of vascular smooth muscle cell function under cyclic mechanical loading in a polyurethane scaffold with optimized porosity. Acta Biomater..

[35] Hollister SJ, Maddox RD, Taboas JM (2002). Optimal design and fabrication of scaffolds to mimic tissue properties and satisfy biological constraints. Biomaterials.

[36] Li MY (2005). Electrospun protein fibers as matrices for tissue engineering. Biomaterials.

[37] Chen H (2017). Tailoring surface nanoroughness of electrospun scaffolds for skeletal tissue engineering. Acta Biomater..

[38] Zamani F, Amani-Tehran M, Latifi M, Shokrgozar MA (2013). The influence of surface nanoroughness of electrospun PLGA nanofibrous scaffold on nerve cell adhesion and proliferation. J. Mater. Sci. Mater. Med..

[39] Zhou Q (2015). Engineering aligned electrospun PLLA microfibers with nano-porous surface nanotopography for modulating the responses of vascular smooth muscle cells. J. Mater. Chem. B.

[40] Perez-Puyana V (2021). Fabrication of hybrid scaffolds obtained from combinations of PCL with gelatin or collagen via electrospinning for skeletal muscle tissue engineering. J. Biomed. Mater. Res. Part A.

[41] Davidenko N, Campbell JJ, Thian ES, Watson CJ, Cameron RE (2010). Collagen-hyaluronic acid scaffolds for adipose tissue engineering. Acta Biomater..

[42] Montanheiro TL (2019). Enhanced water uptake of PHBV scaffolds with functionalized cellulose nanocrystals. Polym. Test..

[43] Lee SJ (2008). Development of a composite vascular scaffolding system that withstands physiological vascular conditions. Biomaterials.

[44] Camasão DB, Mantovani D (2021). The mechanical characterization of blood vessels and their substitutes in the continuous quest for physiological-relevant performances. A critical review. Mater. Today Bio.

[45] Semmler L, Weberruß H, Baumgartner L, Pirzer R, Oberhoffer-Fritz R (2020). Vascular diameter and intima-media thickness to diameter ratio values of the carotid artery in 642 healthy children. Eur. J. Pediatr..

[46] Wise SG (2011). A multilayered synthetic human elastin/polycaprolactone hybrid vascular graft with tailored mechanical properties. Acta Biomater..

[47] Medina-Leyte DJ (2020). Use of human umbilical vein endothelial cells (HUVEC) as a model to study cardiovascular disease: a review. Appl. Sci..

[48] Pham QP, Sharma U, Mikos AG (2006). Electrospun poly(ε-caprolactone) microfiber and multilayer nanofiber/microfiber scaffolds: characterization of scaffolds and measurement of cellular infiltration. Biomacromol.

[49] Soliman S (2010). Multiscale three-dimensional scaffolds for soft tissue engineering via multimodal electrospinning. Acta Biomater..

[50] Hotaling NA, Bharti K, Kriel H, Simon CG (2015). DiameterJ: A validated open source nanofiber diameter measurement tool. Biomaterials.

