# Supplementary material for: Extracellular matrix-derived and low-cost proteins to improve polyurethane-based scaffolds for vascular grafts
Source: Sci Rep. 2022 Mar 28;12:5230. doi: 10.1038/s41598-022-09040-z (PMC8960935; doi:10.1038/s41598-022-09040-z)
Supplement: Supplementary file 1 — Supplementary Information 1. [file 41598_2022_9040_MOESM1_ESM.docx]

Supplementary Information

Extracellular matrix-derived and low-cost proteins to improve polyurethane-based scaffolds for vascular grafts

**Isabella C. P. Rodrigues**^1,2^**, Éder S. N. Lopes**^2^*, **Karina D. Pereira**^1,3^**, Stephany C. Huber**^4^**, André Luiz Jardini**^5^**, Joyce M. Annichino-Bizzacchi**^4^**, Augusto D. Luchessi**^1,3^**, and Laís P. Gabriel**^1,^**

^1^ School of Applied Sciences, University of Campinas, Limeira, São Paulo, Brazil

^2^ School of Mechanical Engineering, University of Campinas, Campinas, São Paulo, Brazil

^3^ Institute of Biosciences, São Paulo State University, Rio Claro, São Paulo, Brazil

^4^ Hematology and Hemotherapy Center, University of Campinas, Campinas, São Paulo, Brazil

^5^ School of Chemical Engineering, University of Campinas, Campinas, São Paulo, Brazil

* Correspondence to: Éder Sócrates Najar Lopes (E-mail: [esnlopes@unicamp.br](mailto:esnlopes@unicamp.br); Telephone: +55 (19) 3521-0017; Street address: Rua Mendeley, 200, Campinas 13083-860, SP, Brazil)

** Correspondence to: Laís Pellizzer Gabriel (E-mail: [lgabriel@unicamp.br](mailto:lgabriel@unicamp.br); Telephone: +55 (19) 3701-6708; Street address: Rua Pedro Zaccaria, 1300, Limeira 13484-350, SP, Brazil)

**Table S1.** Mechanical properties of scaffolds. Statistical differences were analyzed via one-way ANOVA with a Bonferroni post-test (a,b,c denotes a significant difference of p<0.05 between scaffolds).

| **Scaffold** | **Stiffness (kPa)** | **Mechanical strength (kPa)** | **Elongation at break (%)** |
| --- | --- | --- | --- |
| **PU** | 194 ± 19 ^a^ | 1406 ± 136 ^a^ | 1133.3 ± 65.4 ^a^ |
| **PU-Col-El** | 192 ± 56 ^a^ | 546 ± 88 ^b^ | 375.2 ± 26.2 ^b^ |
| **PU-Gel** | 193 ± 17 ^a^ | 624 ± 76 ^b^ | 909 ± 121.9 ^c^ |





**Figure S1.** MTT assay for the PU, PU-Col-El, and PU-Gel scaffolds. Statistical differences were via two-way ANOVA with a Bonferroni post-test (a,b denotes significant difference of p<0.05 between scaffolds; and 1,2 denotes significant difference of p<0.05 between times).
